# Understanding the interactions between *Eimeria* infection and gut microbiota, towards the control of chicken coccidiosis: a review

**DOI:** 10.1051/parasite/2021047

**Published:** 2021-06-02

**Authors:** Thabile Madlala, Moses Okpeku, Matthew Adekunle Adeleke

**Affiliations:** 1 Discipline of Genetics, School of Life Sciences, College of Agriculture, Engineering and Science, University of KwaZulu-Natal, Westville P/Bag X54001 Durban 4000 South Africa

**Keywords:** Coccidiosis, *Eimeria*, Chickens, Gut microbiota, Probiotics, Phytochemicals

## Abstract

The gastrointestinal tract in poultry harbours a diverse microbial community that serves a crucial role in digestion and protection. Disruption of the gut environment due to *Eimeria* spp. parasite infection causes an imbalance in intestinal homeostasis, driving the increment of pathogens such as *Clostridium* species. Coccidiosis infection affects the composition and integrity of gut microbiota, resulting in elevated susceptibility to diseases that pose a serious threat to the overall health and productivity of chickens. Anticoccidial drugs have proven effective in curbing coccidiosis but with concerning drawbacks like drug resistance and drug residues in meat. The exploration of natural alternative strategies such as probiotics and phytochemicals is significant in controlling coccidiosis through modification and restoration of gut microbiota, without inducing drug resistance. Understanding the interaction between *Eimeria* parasites and gut microbiota is crucial for the control and prevention of coccidiosis, and the development of novel alternative treatments.

## Abstract (fr)

Le tractus gastro-intestinal de la volaille abrite une communauté microbienne diversifiée qui joue un rôle crucial dans la digestion et la protection. La perturbation de l’environnement intestinal due à l’infection par le parasite *Eimeria* spp. provoque un déséquilibre de l’homéostasie intestinale, entraînant une prolifération des agents pathogènes tels que les espèces de *Clostridium*. L’infection par la coccidiose affecte la composition et l’intégrité du microbiote intestinal, ce qui entraîne une sensibilité élevée aux maladies qui ont un impact sérieux sur la santé globale et la productivité des poulets. Les médicaments anticoccidiens se sont avérés efficaces pour freiner la coccidiose, mais avec des effets négatifs inquiétants comme la résistance aux médicaments et la présence de résidus de médicaments dans la viande. L’exploration de stratégies alternatives naturelles telles que les probiotiques et les composés phytochimiques est importante pour le contrôle de la coccidiose par la modification et la restauration du microbiote intestinal, sans induire de résistance aux médicaments. Comprendre l’interaction entre les parasites *Eimeria* et le microbiote intestinal est crucial pour le contrôle et la prévention de la coccidiose et le développement de nouveaux traitements alternatifs.

## Introduction

Coccidiosis is the most significant ubiquitous disease in the chicken industry globally caused by obligate intracellular parasitic protozoa belonging to the genus *Eimeria*. It currently results in great economic losses exceeding USD 3 billion yearly due to loss of productivity, high mortality and high treatment costs to control the disease [76, 93]. The disease is characterised by the colonisation and overcrowding of intestinal mucosa of chickens by *Eimeria* spp., compromising chicken wellbeing and productivity. Infections caused by *Eimeria* can easily be transmitted between hosts by the direct faecal-oral route through ingestion of sporulated oocysts from contaminated feed or litter [27, 45]. Seven recognised *Eimeria* species cause coccidiosis at varying degrees of severity in chickens [63]. These species include *E. acervulina, E. brunetti, E. maxima, E. mitis, E. necatrix, E. praecox,* and the most prevalent *E. tenella* [75]. Each species has its own host-specific spectrum, targeting specific sites in the gut and posing severe damage to the host’s absorption capability and growth [37]. Turk [80] reported on colonisation sites preferred by *Eimeria* spp., with the upper small intestine (jejunum and duodenum) mainly infected by *E. acervulina, E. necatrix, E. maxima* and *E. praecox*. *Eimeria tenella, E. mitis,* and *E. brunetti* infections occur in the caecum, ileum and colon, where the reaction of intestinal lymphoid tissue and intestinal damage by *E. tenella* results in significant blood loss at the acute stages of infection, observed as haemorrhagic patches, an increase in caecal protein and DNA which affects chicken weight gain and reduction in muscle mass [21, 44].

*Eimeria* infection destroys host mucosal cells resulting in elevated cell permeability, nutrient and plasma protein leakage, impaired digestion, and protein absorption, contributing to clinical and subclinical effects of coccidiosis [54, 81, 90]. This compromises chicken wellbeing as it disrupts host gut homeostasis, causing significant malabsorption, reduced feed conversion and weight gain, and overall decreased productivity in chickens [44, 63]. The severe intestinal damage caused by *Eimeria* colonisation not only affects epithelial cells, but it causes great disruption of gut microbial communities in the gastrointestinal tract (GIT), promoting colonisation and proliferation of other pathogens such as *Clostridium perfringens*, causing susceptibility of infected chickens to secondary diseases, thus increasing chicken mortality [3, 27, 49]. *Eimeria* invasion results in an imbalance in the gut microbial community known as dysbiosis [18]. The GIT consists of diverse and significant microbes that aid in the nutrition and proper development of chickens. The control of these diseases involves high costs of treatment strategies. This has led to the exploration of various coccidial treatment strategies over the years, such as implementing chemoprophylaxis and live-attenuated vaccines. However, these strategies have proven to be effective with significant drawbacks, including drug resistance and possible parasite reversion to its virulent state. This review aims to understand the interaction between *Eimeria* infection and gut microbiota. Alternative strategies that are safe, antibiotic-free, and cost-effective to control coccidiosis in poultry are also reviewed.

## The function of gastrointestinal gut microbiota

The GIT of the chicken serves a critical role in digestion and protection. It is crucial in converting ingested feeds into nutrients essential for maintenance, growth, and reproduction [6]. It also aids in developing the immune response needed to prevent intestinal colonisation by pathogenic or opportunistic microorganisms through pathogen exclusion [30, 76, 79, 87]. The GIT comprises a complex and diverse microbiota in chickens, including bacteria, viruses, archaea, and fungi. Bacteria are predominant in the GIT and have a beneficial symbiotic interaction with the host, crucial for chicken nutrition, health and product ion [70]. These microorganisms attach to the epithelial wall and trigger the immune system’s maturation consisting of the mucus layer, epithelial monolayer, and other immune cells. The layers form a protective barrier to combat colonisation by opportunistic bacteria [6]. They also produce vitamins (vitamin K), short-chain fatty acids (acetic acid), organic acids (lactic acid) and other complex compounds that provide nutrients and energy essential for the nutrition and protection of the animal [76].

Microbes located in the GIT mainly maintain homeostasis of the intestinal mucosa by digestion of food sources, providing the energy needed to induce the intestinal immune system to fight against aggressions of other microorganisms [14, 86]. A standard or balanced gut microbiota reduces host susceptibility to pathogenic parasites like *Eimeria spp.* [30]. Commensal bacteria regulate nutrient absorption and protect the host by preventing pathogen colonisation through competitive exclusion [89]. They also regulate immune activity by controlling mediators secreted by the mucosa membrane, triggering helper cells [61, 79]. A fully developed immune system secretes proteins such as immunoglobin A (IgA), essential for regulating bacterial composition in the gut [70].

Microbiota located in the caecum or colon (distal gut), such as Ruminococcaceae, produces energy and nutrients through the degradation of substrates such as non-starch polysaccharides to simple sugars using hydrolytic enzymes [8]. *Faecalibacterium* aids in the fermentation of these sugars resulting in the production of substances like short-chain fatty acids (SCFAs), i.e. butyrate and essential amino acids available for host consumption [87]. Production of butyrate in chickens is crucial for reducing chronic inflammation and relieving the severity of *E. tenella* infection [14]. The presence of SCFAs not only serves as the energy and carbon source for broilers, but also aids in the regulation of blood flow, which stimulates cell growth in the intestinal lining [16]. The degradation or fermentation of carbohydrates and polysaccharides releases easily accessible energy to other microorganisms, allowing further metabolic processes to occur [60].

### Normal gut anatomy and microbiota

For chickens to effectively utilize end-products resulting from metabolic processes, a balanced microbial community is crucial to facilitate internal interaction between host and diet [86]. The chicken GIT is divided into sections consisting of diverse and unique microflora playing different metabolic functions; these sections include the crop, proventriculus, gizzard, small (jejunum and duodenum) and large intestines (ileum, caecum and colon) [61, 67, 80]. These sections harbour diverse communities of commensal, symbiotic and pathogenic microorganisms, e.g. crop, duodenum, and gizzard share almost similar microbiota composition (about 99% *Lactobacilli* population) [24]. The bacterial composition and diversity within the GIT influence intestinal functions such as digestion and nutrient absorption. A balanced chicken GIT is predominated by Firmicutes, Tenericutes, Bacteroidetes, and Proteobacteria [8]. These commensal bacteria are located in the crop, with *Lactobacillus* exhibiting the highest diversity. They aid in the hydrolysis of starch and fermentation of lactate [10]. They are also present in high abundance in the ileum, facilitating nutrient absorption [66]. It has been observed that even though the composition of gut microbiota located in the ileum is more distinct and dominated by *Lactobacillus, C. arthromitus, Enterococcus,* and *Clostridium*, it is also less stable compared to other sections of the GIT (duodenum and the jejunum) [51, 60].

The caecum is known to have the most remarkable taxonomic diversity and shelter more diverse microflora, including anaerobes, e.g. *Clostridium, Bacteroides,* Proteobacteria, Actinobacteria and Firmicutes [67, 76, 95]. As broilers advance in age, caecal gut microbiota undergoes significant changes resulting in a richer and stable microbial community [25]. These microbes play a crucial role in the fermentation and digestion of complex substrates such as cellulose, starch and other polysaccharides [81]. Microbial richness and diversity can be observed in the caecum during the second week, where most microbes from the phylum Proteobacteria are replaced and dominated by Firmicutes-Lachnospiraceae and Ruminococcaceae [40]. Previous research shows that changes in caecal microbial communities are likely due to microbe transfer through direct contact with the adult hen to offspring and chicks ingesting the mother’s faecal material [80]. During the direct transfer, hens are significant donors of Bacteroidetes and Actinobacteria [40]. Development and changes in gut microbiota often compromise the immune system and health of chicks, leaving them susceptible to various bacterial and parasitic infections, e.g. *Salmonella, C. perfringens*, and *Eimeria.* Direct contact of offspring with older hens plays a vital role in microbial development and maturation regardless of environmental factors (feed and litter) [61]. Since microbiota becomes more diverse and stable as birds grow, changes observed in the gut, whether genetically transmitted by breeder birds or influenced by the environment, often result in a build-up of harmful bacteria which disrupts gut microbiota over beneficial ones [3, 78].

### Effect of coccidian (*Eimeria*) infection and pathogenicity in chicken gut microbiota

*Eimeria* infection has a severe impact on broilers resulting in known physical symptoms such as depressed growth performance, decline in body weight gain, and low production [47, 95]. These clinical effects remain a leading contributor to economic losses, with the poultry industry suffering high losses due to treatment and chicken mortality. The external expression of these clinical symptoms is due to internal disruption of enterocytes and intestinal epithelial cells, causing unevenness in intestinal homeostasis and elevated susceptibility risk of the host to other diseases [29, 70]. During an *E. tenella* infection, the presence of severe intestinal epithelial injuries negatively impacts the proliferation and growth of resident bacteria [14]. Cytokines such as IL-17A, IL-10, IL-1β and IFN-γ are secreted, providing pathways that favour proliferation and survival of pathogens [85]. *Eimeria* uses these pathways (Toll-like receptors-TLR-2 and TLR 6) to exploit the IL-10 mRNA production, invade the host immune system and complete its life cycle [68]. The highjacking of pathways by *Eimeria* poses a great threat to the health of chickens, affecting proper production and utilisation of nutrients.

**Figure 1:**
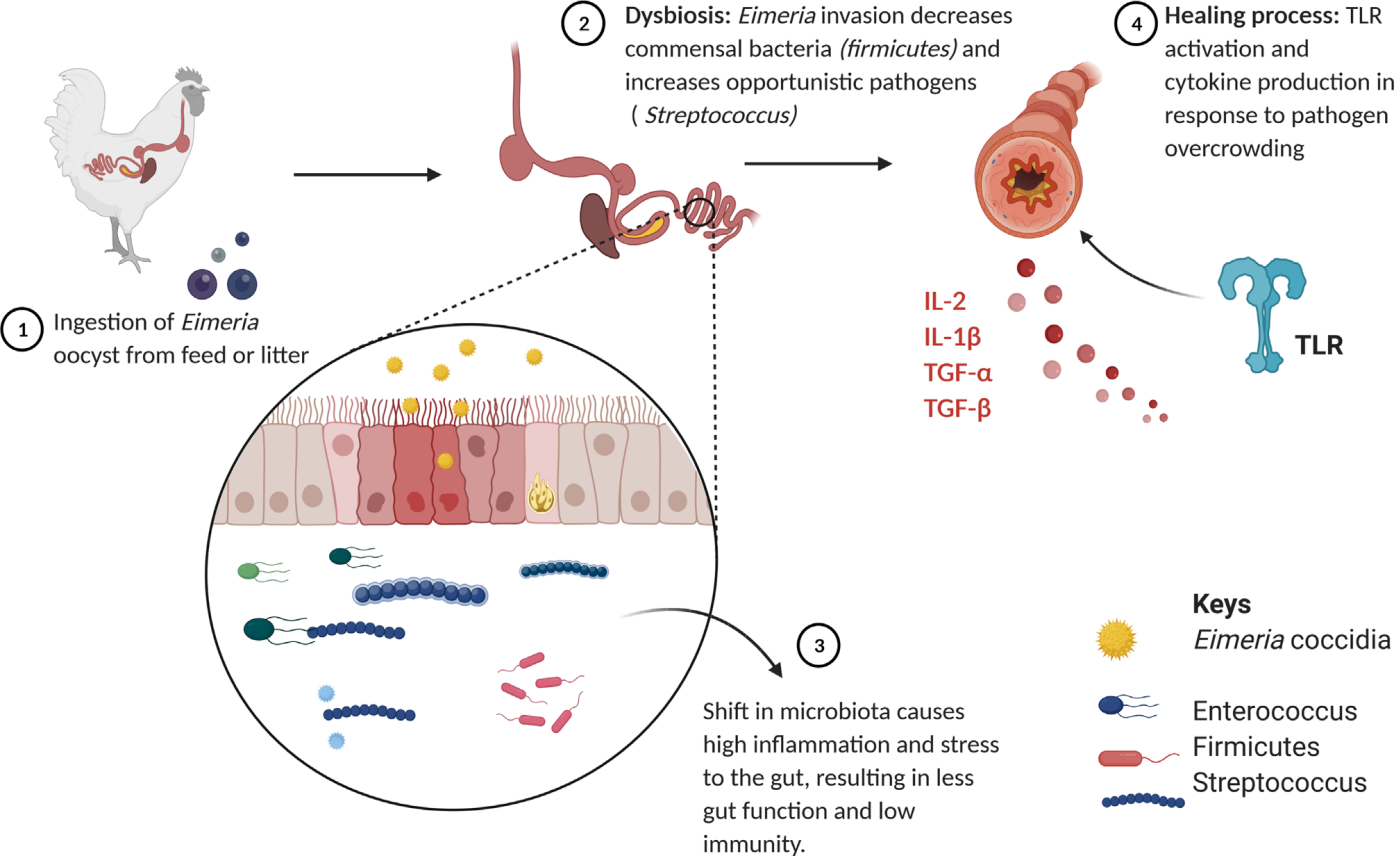
Impact of coccidiosis on bacterial species in the gastrointestinal tract of chickens.

*Eimeria* infection destroys the structure of caecal tissues and intestinal lining, causing a disturbance in the gut microbial community, known as dysbiosis [18]. Dysbiosis is exhibited by a significant fluctuation of beneficial bacteria, while harmful bacteria accumulate to the extent of becoming a potential threat to the host, causing an imbalance in host homeostasis [17]. An overview of *Eimeria* infection impact on bacterial species of the gut is shown in Figure 1. Zhou et al. [95] reported that *E. tenella* infection altered composition and diversity of caecal microbiota, significantly reducing Proteobacteria and Firmicutes *(Enterococcus).* It also causes shifted gut microbiota, reducing the caecal microbial diversity of chickens [87]. *Eimeria* infection affects the bird’s ability to digest nutrients by reducing intestinal barrier function, causing bacterial translocation, affecting bacteria-dependent metabolic processes in the GIT [70]. Latorre et al. [41] reported that necrotic enteric diseases like coccidiosis affected the diversity and composition of the bacterial community in the GIT, reducing bacterial species such as Firmicutes, (mainly Ruminococcaceae) and SCFA-producing bacteria. *Eimeria* infection can decrease the frequency of immune-modulating bacteria (i.e. *Candidatus savagella,* Ruminococceae), while the abundance of bacteria (*Clostridium* spp.) that cause lesions and coccidia replication increases, causing severe damage to the mucosa [27]. The impact of coccidiosis bacterial species is summarised in Figure 1.

A study by Macdonald et al. [48] examining the effect of *Eimeria* infection (*E. tenella*) on caecal microbiome diversity revealed that *Eimeria* infection did not affect microbial alpha diversity but induced significant changes observed by severe lesion score damage in the caecum and reduction in the microbial population of Bacillales and Lactobacillales, suggesting that changes in abundance of the bacterial community may contribute to the severity of pathology and variation observed in tissue damage. Co-infections with *Eimeria* and *C. perfringens* also result in severe necrotic enteritis lesions with a significant increase of *Clostridium sensu stricto 1*, *Escherichia, Shigella,* and *Weissella*, and reduction in the *Lactobacillus* population located in the jejunum [10, 84]. Reduction of commensal bacteria such as *Firmicutes (Lactobacillus)* affects microbial diversity. It disrupts crucial metabolic processes that provide energy and carbon sources of the host [89]. These bacteria aid in converting glucose to lactic acid, lactate, acetic acid, ethanol and CO_2,_ which serves as an energy powerhouse for the host [22]. *Proteobacteria (Campylobacter)* prevalence assists hydrogenases in hydrolysing indigestible sugars, namely; polysaccharides, oligosaccharides, and disaccharides aiding in the production of short chain fatty acids (SFCAs) [9]. Depletion of these bacteria due to *E. tenella* infection results in the build-up of complex compounds like uric acid and non-starch substrate indigestion. These compounds can be toxic to the host, hindering the production of amino acids essential for facilitating the production of SCFAs absorbed by enterocytes [16].

Some reports show that *Eimeria* infection alone has little to no effect on α-diversity and caecal microbiota. However, co-infections with *Eimeria*, and other predisposing factors (*C. perfringens*) affecting the GIT can reshape or shift intestinal microbiota. Microbial shift results in loss of diversity and depletion of crucial gut microbiota, e.g. *Lactobacillus*, while favouring overgrowth of pathogenic bacterial strains (*Clostridium, Salmonella* and *Weissella*) that affect growth and health of the birds [28, 40, 50, 56, 93]. Supplementation of deficient bacteria to control *Eimeria* challenge is crucial, by the administration of natural dietary feeds/additives such as probiotics, prebiotics, and phytochemicals, which are able to improve function and the abundance of microbes, thus modulating gut microbiota.

## Dietary supplementation for manipulating the chicken gut microbiota

Various strategies are currently being employed to combat the severity and spread of coccidiosis in chickens. Some of these control strategies include the use of live vaccines, therapeutic antimicrobial growth promoters (AGPs) and anticoccidial drugs (such as ionophore-based prophylactic drugs) produced from synthetic chemicals and fermentation methods [5, 34, 57]. The use of these control strategies in previous years has proved effective in controlling the spread of *Eimeria* infections; however, continuous exposure of animals to live vaccines and AGPs has been discouraged as it poses negative impacts on food animals and indirectly public health concerns to humans [88].

The use of live vaccines and AGPs (ionophores) hinders host colonisation by parasites through disruption of the parasite’s replication cycle, interference of ion transfer, and other biochemical pathways in the plasma membrane of the parasite, leading to parasite death. Regardless of the effectiveness of these controls, continuous exposure of poultry to such treatments has resulted in the development of drug resistance and detection of traces of chemicals or compounds in poultry products (meat and eggs) as residuals that could potentially be harmful to humans when consumed, raising concerns and prompting calls for their discontinuation [5, 57, 64]. As a result, a diversion is observed in research to explore various antibiotic-independent alternative strategies that are safe, antibiotic-free, and cost-effective to ameliorate coccidiosis in poultry, e.g. the use of probiotics, prebiotics, organic acids, phytobiotics and other natural additives, such as diet supplements in broiler feed [1, 23, 94]. Coccidiosis greatly impacts gut barrier function, which is crucial for optimal health and defence of host; hence, an ideal feed additive should promote efficient barrier function of the gut. Chen et al. [15] identified potential biomarkers (IL-8 and TGF-β) for barrier failure in broilers relevant for gut functioning in chickens. Administration of natural feeds such as probiotics should trigger cytokine production (IL-2, TGF-α) to confront coccidiosis.

### Modulation of gut microbiota by probiotics against coccidial infection

Probiotics are live, well-defined non-pathogenic culture of microorganisms ingested by the host, crucial in improving inhabitant intestinal microbiota. These are often bacteria or yeast extracts added to the broiler’s diet to enhance production and animal health by improving the intestinal microbial balance [5]. These bacteria or extracts secrete crucial substances like bacteriocins and organic acids that contribute to health and homeostasis of the intestinal tract. They can protect against the invasion of pathogenic bacteria and coccidiosis by competitive exclusion [12, 28]. Investigating probiotics as potential alternative strategies to alleviate *Eimeria* challenge in broilers is still crucial following previous literature reporting on the benefits of using these natural additives. Probiotics have been reported to exhibit immunomodulation properties by manipulating and modifying gut microbiota, resulting in improved feed conversion ratio (FCR) and performance in broilers [5, 74]. Some of the bacteria used as probiotics are isolated from fermented milk, including *Lactobacillus, Bacillus, Pediococcus, Saccharomyces cerevisiae* and *Enterococcus faecium* [19]. Li et al. [46] reported effective response observed in broilers upon administration of *Lactobacillus*-based probiotics (*L. acidophilus*), which boosted inflammatory response through production of acidophilin and modulated the immune responses (innate and acquired) of poultry, providing a favourable environment for beneficial bacteria [24].

During *Eimeria* infection, the parasites replicate and adhere to receptors located in the intestine epithelial surface. Intestinal compatible probiotic bacteria challenge *Eimeria* parasites in adhering to the intestinal mucosa and absorb receptors in the epithelial cells, preventing invasion [33]. This attachment hinders the perforation and secretion of *Eimeria* sporozoites into the intestinal mucosa, further resulting in reduced proliferation and oocyst shedding [58]. This regulates host intestinal permeability, improves barrier functions of the gut, and balances host gut microbiota [31]. *Eimeria* infection affects intestinal permeability, so the administration of feed additive like probiotics could promote the balance of gut microbiota and regulate the barrier function to prevent the entry of foreign microorganisms in the GIT.

Probiotics improve beneficial bacteria’s ability to adhere to the GIT, enabling them to occupy most of the tract while inhibiting the growth of pathogenic bacteria and parasites [84]. Probiotics employ other competitive exclusion modes of action to reduce parasite colonisation, including competition for sites of attachments, co-aggregation with pathogens and certain gut microbes and production of antimicrobial compounds that stimulate the immune system, e.g. lactic acid, hydrogen peroxide and bacteriocins [2, 38]. These mechanisms promote animal growth and balance in gut health through microbial abundance in the GIT [69]. Competitive exclusion exhibited by probiotics against *Eimeria* species results in improved intestinal health through gut maturation and improved integrity, leading to improved feed digestion and absorption, hence higher body weight gain is often observed in chickens after probiotic treatment [65]. Alagawany et al. [2] reviewed the modes of actions exhibited by probiotics to hinder invasion and proliferation of pathogens such as *Eimeria* and *Salmonella*. The mechanism employed by probiotics involves the production of antibacterial substances that play a role competitively excluding unwanted and harmful bacteria from entering the gut [2, 38]. Probiotics enhance host immune responses by regulating T-helper cells, conferring improved protection against the *Eimeria* challenge [26]. Research has revealed probiotics as a promising alternative to antibiotics; however, probiotic incorporation is highly dependent on chicken diet (optimal dose) and selection of appropriate strains of probiotic microbes to exhibit effective action against specific pathogens [18].

Probiotics are also crucial for stimulating the proliferation of the intestinal epithelium, which regulates the mucosal barrier by mucin in the intestinal wall of broilers, lowering bacterial diversity [19, 26]. The use of probiotics such as *Bacillus subtilis* as dietary supplementation modifies gut microbiota composition by improving growth performance and nutrient digestion in broilers through an increased abundance of *Bacteroidetes* and other commensal bacteria, such as *Ruminococcus* [46]. Similar findings were reported by Wang et al. [84], where *B. subtilis* was confirmed to reduce microbial diversity in the caecum by altering microbial community and elevating predominant species. The abundance of these bacteria aids in the breakdown of indigestible fibres, releasing substances such as butyrate and SCFAs, which are essential for extra nutrients and energy for broilers [24, 44]. Probiotics mainly target microorganisms in the small intestines, where peak nutrient absorption is observed [84]. The presence of probiotics (i.e., *Lactobacillus*) in different sites in the GIT increases nutrient utilisation, hindering intestinal colonisation and intestinal lesions caused by *E. tenella* invasion [65]. In evaluating the immune effect of infection in broilers, Awais et al. [5] revealed that *Lactobacillus* and *Saccharomyces-*based probiotics enhanced immunological and performance capabilities in broilers challenged with *Eimeria*. This property enables probiotics to protect against *Eimeria* infections, thereby reducing coccidiosis prevalence.

*Lactobacillus-*based probiotics have been reported to exert vital anticoccidial properties against *E. tenella* infections [5, 14]. These probiotics exhibit growth-promoting effects that promote enhanced cellular and humoral immune responses, restricting the invasion of the GIT by *Eimeria spp.* [44]. They also act as antagonists by producing cytokines and local antibodies (immunoglobulin A-IgA), which are crucial for confronting coccidiosis, i.e. interleukin-2 (IL-2), interferon‐γ (IFN‐γ) and IL-6, and stimulate local cell-mediated immunity inhibiting invasion by *E. acervulina* [7, 24, 26, 74]. Cytokines are natural proteins essential for stimulation and regulation of immunity against infectious diseases. These substances also hinder the secretion of siderophores, restricting iron availability to facilitate parasite invasion [5]. Probiotics also protect against *Eimeria* by stimulating mucosal immunity and reducing oocyst shedding [45].

Chen et al. [13] previously showed that a probiotic mixture containing four strains of lactic acid bacteria (*L*. *acidophilus*, *L. fermentum*, *L. planetarium,* and *E. faecium*) was effective in reducing intestinal ulcers observed in broilers due to *E. tenella* infection, and significantly influenced the level of expression of specific genes crucial for inflammation, e.g. cytokines [26]. *Lactobacillus-*based probiotics reduced levels of caecal gene expression of cytokines favouring inflammation (e.g. interleukin (IL)-1β and IL-6) and interferon (IFN)-γ, while anti-inflammation cytokine, i.e. anti-IL-10, was increased [4, 13]. Probiotics’ capability to exert stimulating effects on the immune system, while altering the gut microbiota profile [50], cement their consideration as suitable candidates to control coccidiosis. More research has been done to determine the effectiveness of other probiotic microflora against coccidiosis. *Enterococcus faecium* increased the abundance of beneficial bacteria, while also modulating the composition of intestinal microflora. *Enterococcus faecium* and *Pediococcus* supplementation in broilers infected with *E. acervulina* exhibited immunomodulatory effects through increased production of cytokines aiding in the modulation of anti-inflammatory cytokines and other immune mediators, including IL-1β, IL-6, IL-10, and IFN-γ, reducing the severity of intestinal lesions caused by the infection [43, 77, 87]. Although in-depth information on anticoccidial properties of some probiotics has been reported, efforts in exploring other microflora in the gut with potential to induce protection against necrotic enteritis infectious diseases is still required.

Even though probiotics effectively control coccidiosis, research has revealed that simultaneous inclusion of prebiotics with probiotics as poultry feed additives can enhance the viability of probiotic microorganisms through a synergistic effect [14, 44]. Pineda-Quiroga et al. [59] showed that supplementation with synbiotics in the broiler diet conferred modulatory effect on microbiota, while enhancing various pathways (i.e. starch and sucrose metabolism) without interfering with the biological role of caecal microbiota compared to when pre- and probiotics are administered independently. Similarly, the combination of pro- and prebiotics allows prebiotics to activate probiotics for effective modulation of the metabolic response in the gut against infections, while maintaining gut microbiota integrity and inhibition of potential pathogens present in the digestive system [73]. Muthamilselvan et al. [53] further reviewed anticoccidial action of herbal remedies against coccidiosis, noting close interactions between prebiotics and probiotic microorganisms inhabiting the gut. Prebiotics are non-digestible feed additives that contain natural dietary fibres such as fructooligosaccharides (FOS), inulin, β-glucans, and mannooligosaccharides (MOS), which are crucial in inducing development or activity of commensal bacteria in the gut, excluding invasion of the harmful pathogens [23, 55, 56]. Accumulation of gut probiotics mediated by prebiotics suppresses pathogens and significant improvement of poultry’s immune responses [88]. Even though *Eimeria* infections drastically reduce majoring of commensal bacteria, studies have found a mild increase of some *Firmicutes* (Ruminococcaceae) in infected chickens after *E. tenella* infection [14]. Microbe recovery after infection allows fast restoration of GIT mucosa, maintaining gut homeostasis.

### Stimulation and stabilisation of gut microbiota through phytobiotics

The use of plant-based phytochemicals has been associated with their antimicrobial and antiparasitic properties that enhance protective immunity in livestock infected by coccidiosis and other necrotic enteritis diseases [32]. Phytobiotics include various products synthesized and extracted from plants such as herbs, essential oils, and oleoresins [52]. Supplementation of these natural products as animal feeds has been favoured because they are safe, cheap, readily available in nature and effective against various diseases [1, 57]. Some of these natural foods and herbs improve immunity and animal resistance to disease. The mechanisms of action utilized by phytochemicals in controlling infections include interference with the life cycle of *E. tenella* by hindering oocyst sporulation, invasion of sporozoites and the maturation of schizonts [93]. They have been reported to impair *Eimeria spp*. in early development stages by reducing cell wall degradation and inducing oxidative stress, preventing invasion [20, 53]. Similar findings were reported by Jiao et al. [35] where administration of *Artemisia annua (A. annua) and Artemisinin* was confirmed to interfere with the *Eimeria* life cycle by producing reactive oxygen species that are effective in inhibition of oocyst sporulation and formation of the parasite cell wall.

Research done to date on phytonutrients such as *Capsicum annuum* (pepper), *Curcuma longa* (turmeric), *Lentinus edodes* (shiitake mushroom), and *Carthamus tinctorius* (safflower) revealed promising inhibitory properties and defence mechanisms against infections in chickens, promoting enhanced gut health and immune system [39, 82]. There is a wide range of commercialised plant-derived compounds available, and their effect on chicken health thus far has been promising. Some phytochemicals and their effect on immune response and gut health are presented in Table 1. Administration of phytochemicals in chickens challenged with *Eimeria* alters and stabilises the intestinal microbiota by shifting the composition of beneficial bacteria and reducing microbial metabolites in the gut, improving gut health [90]. Kim et al. [39] further confirmed that the administration of capsicum and curcumin longa oleoresins affected the microbial population by increasing abundance of *Lactobacillus* and operational taxonomic units, while reducing the population of *Selenihalanaerobacter* in two chicken breeds (Ross and Cobb). Cinnamaldehyde and Oleoresins (Capsicum and Turmeric) have been reported to regulate host immunity against *E. tenella* through elevation of T helper cells and cytokines (FN-γ and IL-6) and body weight gain in poultry [42]. A combination of curcuma and capsicum exert synergistic effects against coccidiosis by enhancing innate immunity. The mechanisms exhibited by phytobiotics on the regulation of the life cycle of *Eimeria* and growth regulation of gut bacteria make phytochemicals best suited for supplementation in the poultry diet at an early stage of development.

**Table 1:** Different phytochemical additives against coccidiosis in poultry.

Phytochemical	Effect on gut health and immune response
Areca Nut (*Areca Catechu L.*)	Reduced caecal lesion scores. Enhanced immunity through the production of interleukin 2 (IL-2) [83]
Bidens Pilosa	Disrupted life cycle of *E. tenella* [12, 91] Enhanced T-cell mediated immunity [14, 92]
Carvacrol	Increased growth performance and intestinal barrier function [20 ltered gut physiology Interference with the life cycle of *E. tenella* by destroying sporozoite membrane [42]
Curcumin	Maintenance of gut integrity [88] Interference with life cycle by inhibiting *E. tenella* sporozoites [11]
Cinnamaldehyde	Improved chicken growth performance and altered caecal microbiota composition [90]
Garlic (*Allium Sativum*)	Increased resistance to experimental *Eimeria acervulina* infection [62, 71] Induced alterations in broiler intestinal microbiota

Similar results were found by Chowdhurya et al. (2018) when evaluating the effect of three essential oils, i.e. cinnamon bark oil (CNO), clove bud oil (CLO), and ajwain seed oil (AJO), where the abundance of *Escherichia coli* in pre-caecal contents decreased in groups given CNO, but did not affect *Lactobacillus* spp. in any diets. Based on a range of activities exerted by phytobiotics in chickens, it can be suggested that the primary mode of action for most phytobiotics focuses on altering the gut environment and intestinal morphology, providing protection and enhanced resistance to infections when fed to healthy poultry [32, 36]. From the findings mentioned above, the simultaneous use of feed additives may be beneficial to the host by providing different functions, such as maintaining chicken gut health and targeting different aspects of animal physiology to boost antimicrobial activity against diseases. Research has also shown that combined supplementation of phytochemicals exerts boosted anticoccidial effects in broilers compared to when administered on their own [20, 72].

## Conclusion and future recommendations

Coherent interaction between gut microbiota and the host is crucial for the normal functioning and health of poultry. Presence of a coccidian infection (i.e. *Eimeria* or *Isospora*) in chickens compromises gut microbiota resulting in an imbalance of the microbial communities, affecting their active role within a host. Supplementation with natural alternatives such as probiotics and phytochemicals remains the most favoured strategy currently to combat coccidiosis, without the effect of drug resistances threatening biosecurity. Manipulation of gut microbiota by introducing natural additives at an early stage of development may improve the immune system of chickens against *Eimeria* infections. Since these additives are chemicals and strains fermented by bacteria, they can persist in the GIT as a beneficial gut microbiome for a long time, without posing any adverse effect on broilers. Thus, the use of natural alternatives (feed additives) is recommended for fast GIT restoration and modulation of gut microbiota in response to coccidian challenges, while improving growth performance and overall health of broilers. Significant progress has been made in discovering alternative strategies to combat coccidiosis; however, further studies focusing on joint interaction of feed additive with host gut microbiota towards *Eimeria* challenge are still required to provide efficacious treatment measures.

## Author contributions

T Madlala wrote the manuscript. M Okpeku and MA Adeleke supervised the work and corrected the manuscript. All authors approved the manuscript.

## Declaration of conflict of interest

The authors declare that they have no conflict of interest.
